# Human Toxocariasis in Portugal—An Overview of a Neglected Zoonosis over the Last Decade (2010–2020)

**DOI:** 10.3390/idr13040086

**Published:** 2021-11-04

**Authors:** Ana Margarida Alho, Pedro Manuel Ferreira, Isabel Clemente, Maria Amélia Afonso Grácio, Silvana Belo

**Affiliations:** 1 Public Health Unit USP Francisco George, Primary Medical Healthcare Cluster Lisbon North (ACES Lisboa Norte), Largo Professor Arnaldo Sampaio, 1549-010 Lisboa, Portugal; margarida.alho@arslvt.min-saude.pt; 2 Global Health and Tropical Medicine, GHTM, Instituto de Higiene e Medicina Tropical, IHMT, Universidade Nova de Lisboa, UNL, Rua da Junqueira 100, 1349-008 Lisboa, Portugal; pedroferreira@ihmt.unl.pt (P.M.F.); isabel.clemente@outlook.com (I.C.); mameliahelm@ihmt.unl.pt (M.A.A.G.)

**Keywords:** *Toxocara*, epidemiology, Portugal, positivity, children, public health

## Abstract

Toxocariasis is one of the most widespread and important zoonotic parasitic diseases, although neglected. Data regarding human *Toxocara* infection in Portugal are almost absent. This article gives an overview of the situation of toxocariasis in Portugal over the last decade based on casuistic data. A total of 846 serum samples from individuals suspected of toxocariasis, collected from 2010 to 2020, were analyzed at the Institute of Hygiene and Tropical Medicine. Sera were tested for IgG antibodies to *Toxocara canis* excreted–secreted larval antigens by enzyme-linked immunosorbent assay and counterimmunoelectrophoresis. Positivity was detected in 18.8% (159/846) [CI 95%: 16.3–21.6], with positives detected throughout continental Portugal. Overall, 59.7% of the positives were diagnosed in younger than 20 years (35.2% aged 0–9 years and 24.5% aged 10–19 years). Eosinophilia was the most frequent feature reported (27.7%). Pediatrics (41.5%) and Infectiology (25.8%) were the specialties with the highest number of positives. An average of 77 samples/year were received, recording a maximum positivity in 2012 (41.5%, *n* = 27/65) and a minimum in 2020 (6.4%, *n* = 3/47). These numbers may reflect the effectiveness of current preventive measures, highlighting the need to maintain public awareness to control this helminthozoonosis and promote a higher public health standard.

## 1. Introduction

Toxocariasis is reported to be one of the most widespread zoonotic parasitic infections and listed as one of the five most important neglected diseases by the CDC [1,2]. Humans may become infected by the accidental ingestion of embryonated eggs of the nematodes *Toxocara canis*, *Toxocara cati* and/or congeners, from the water or vegetables or by the ingestion of hypobiotic (arrested) larvae in paratenic hosts [3]. However, the predominant mode of infection is through the ingestion of infective eggs from the environment, by geophagia, a common situation in children [4].

The most widespread zoonotic species is *T. canis*, which is responsible for the majority of human toxocariasis cases. *T. canis* may infect a wide variety of canids (dogs, foxes, wolves, coyotes and jackals), while the cat roundworm, *T. cati*, infects felids as definitive hosts [4]. Humans, rodents, birds, rabbits, cattle, sheep, pigs and poultry may serve as paratenic hosts [5]. Fertilized female worms may release several hundred thousand eggs per day, which under favorable temperature and humidity conditions, will embryonate over weeks [3,4,6].

Although exposure to *Toxocara* spp. infection may be common in humans, a low number of people develop larva migrans and/or clinical manifestations. Recognized human toxocariasis syndromes comprise: visceral larva migrans (VLM), ocular larva migrans (OLM), neural larva migrans (NLM) and covert/common toxocariasis (CT). The severity of the disease is dependent on the parasite burden, the duration of larval migration and on the host immune response [1,7]. VLM has a higher prevalence in young children (aged 1–7 years) and results in hepatitis and pneumonitis, with hepatomegaly, eosinophilia, lymphadenopathy, cough, wheezing, fever, weight loss, diarrhea and vomiting [4,8]. OLM occurs more frequently in older children (aged 5–10 years) and adolescents, resulting in unilateral vision impairment with granuloma formation and retinal damage [8,9]. NLM results in meningoencephalitis and cerebritis, and presents with headache, fever and seizures, mainly in middle-aged people. CT, representing “common toxocariasis” in adults and “covert toxocariasis” in children is similar to VLM but far more common and with mild manifestations.

Most infections are asymptomatic and may go unnoticed, as clinical investigation and diagnostic testing are frequently not conducted [7,10]. A high index of suspicion is, therefore, necessary to establish an early diagnosis. Generally, its diagnosis is based on history (geophagia or consumption of raw/undercooked meat), clinical examination, blood analysis (leukocytosis and eosinophilia) and microscopic examination of tissues (eosinophilic granuloma surrounding degenerated or live roundworm larvae), although detection of larvae in tissues is difficult, invasive and time-consuming. Thereby, diagnosis of human toxocariasis is usually achieved by serology, through enzyme-linked immunosorbent assays (ELISAs) using *Toxocara* excretory/secretory (TES) antigens, sporadically combined with imaging to detect encapsulated larvae in tissues [1,4,11].

Treatment in humans varies, depending on the clinical symptoms and the location of larvae. It is mostly treated with the anthelmintics albendazole (first option) or mebendazole, along with corticosteroids to reduce allergic responses linked to *Toxocara* antigens [7].

As *Toxocara* spp. are such ubiquitous and prolific nematodes, the disease is widespread in many countries, reaching high prevalence, even in economically stable countries. Despite the public health and clinical significance, the true number of cases of toxocariasis is likely to be underestimated due to its non-specific symptoms, lack of adequate surveillance programs and the fact that is a not notifiable disease. Portugal is not an exception with very limited data available. No human seroprevalence surveys have been carried out in Portugal. There is only a report from an official laboratory (INSA) in southern Portugal of 21.9% positive sera for *Toxocara* sp. in 457 suspected human samples from 1995 to 2005 [12]. However, both *Toxocara* spp. are widely distributed in Portuguese domestic and wild carnivores, ranging from 5% in owned dogs to 29% in stray dogs from the North of Portugal [13,14,15,16], 11–38% in stray cats from Lisbon [17,18] and owned and shelter dogs from Évora [19]. It is also prevalent in Iberian wolves (9%) and red foxes (21–37%) from central Portugal [20,21].

The present article evaluates the positivity of serological tests to *Toxocara* based on casuistic data and explores the trends of this disease in Portugal over the last decade.

## 2. Materials and Methods

Samples from individuals suspected of toxocariasis were sent to be tested at the Portuguese Institute of Hygiene and Tropical Medicine (IHTM), Universidade NOVA de Lisboa, one of the reference laboratories for human tropical parasitic diseases in Portugal. Serum samples were obtained from 20 medical specialties from 29 different medical institutions (hospitals, clinics, and primary healthcare centers), located throughout the five regional health administrative units of continental Portugal (North, Central, Lisbon and Tagus valley (LTV), Alentejo and Algarve).

A total of 846 human serum samples collected from January 2010 to December 2020 were tested by ELISA (macro method) and immunoprecipitation assays for toxocariasis, using an excretory–secretory antigen derived from second-stage larvae of *T. canis* (TES) prepared in house according to the De Savigny protocol (1975) [22], obtained from several batches over the decade, and tested and optimized before its application. In brief, serologic levels of immunoglobulins IgG, anti-parasite in this sample, were determined by indirect ELISA using *T. canis* L2 larva antigen (TES) produced in-house. All immunoassays’ procedures were performed according to standardized protocols used in our laboratory [23]. In brief, polystyrene MaxiSorp™ tubes (Nunc-Immuno™ Tubes, Thermo Scientific) were coated with TES (2 µg/mL, 2 mL/tube) diluted in 0.1 M phosphate buffer saline (PBS), pH 7.2, and incubated overnight at 36 °C. Tubes were washed three times with PBS containing 0.05% Tween 20 PBST), followed by incubation of sera diluted at 1:500 (IgG) in PBST (as above). After three washes, the anti-human IgG (Fab specific)-horseradish peroxidase antibody (Sigma-Aldrich^®^, St. Louis, MO, USA) was added in 1:40,000 dilution and revealed by the addition of orthophenyldiamine and H_2_O_2_ (Sigma-Aldrich^®^, St. Louis, MO, USA). Optical density (OD) was measured at 492 nm on the ELISA reader after the addition of 50 µL of 2N HCl. The cut-off was established as the OD mean of the negative controls plus three standard deviations (IgG − OD = 0.500).

The commercial kit ELISA-IgG^®^ (Bioactiva Diagnostica, Bad Homburg, Germany) was used for the detection of IgG anti-*Toxocara* spp. antibodies in samples with doubtful results (i.e., ELISA OD > 0.4500 e OD < 0.500).

To assess specific reactivity of antigen–antibody binding, undiluted serum samples were tested by counterimmunoelectrophoresis (CIE) against TES and *Ascaris suum* crude adult worm’s antigen. CIE was performed in a thin layer of 1.2% agarose gel (A-5304 Sigma-Aldrich^®^) on Sodic Veronal buffer pH 8.2, on a microscope slide. Antigens and serum samples were placed in two pre-done wells, with different sizes (4 mm for sera and 2 mm for TES antigen, with 5 mm between them) and submitted to an electric field (200 V, for 45 min), following incubation overnight at room temperature. Then, slides were placed in a Petri plate with 5% sodium citrate for 3 h, and then with 0.85% NaCl, for 24 h. Slides were dried with filter paper Whatman nº1 and stained with Amidoschwarz 0.1% (Amido Black 10B, Sigma-Aldrich^®^). Results were based on observed precipitation reactive traces.

The protocol was accepted by all the institutions involved. Serum samples were tested exactly at the time they were received in IHTM. All 846 samples belong to different patients, as follow-up analyses were not included in this data. Patients’ demographic and clinical data were obtained by written questionnaire at the 29 medical institutions. Questions include the name of the hospital, medical specialty, sex and age of the patient, as well as major clinical manifestations (abdominal pain, cough/wheezing, elevated total immunoglobulin E, emesis, fever, hepatomegaly/splenomegaly, lymphadenopathy, peripheral blood eosinophilia, persistent diarrhea, unilateral uveitis, weight loss or other). Moreover, main laboratory/imaging findings performed to assess the clinical manifestations were also questioned. Additionally, associated risk factors were asked, such as the consumption of undercooked meat or unwashed vegetables, contact with cats, dogs, or other animals, geophagia habits, or history of playing in soil or sand.

### Data Analysis

Frequency distributions and statistical relationships were analyzed using the statistical software package Stemstat, IBM SPSS Statistics 26^®^ software. Positivity and 95% confidence intervals (CI) were calculated using EpiTools (Agresti-Coull test). The Pearson Chi-square (χ^2^) test was used to compare positivity values. A *p*-value of 0.05 was considered statistically significant.

## 3. Results

### 3.1. ELISA and CIE

Out of the 846 ELISA tested serum samples, 159 were positive (18.8%), 678 negative (80.1%) and nine (1.1%) remained inconclusive, after re-testing with the commercial kit ELISA-IgG^®^. An overall positivity of 18.8% [CI 95%: 16.3–21.6] was found between 2010 and 2020.

By CIE against *A. suum* crude adult worm’s antigen, 73 samples were positive (8.6%) and 773 samples were negative (91.4%). By CIE against TES, 30 samples were positive (3.5%) and 816 samples were negative (96.5%).

High predictive negative values were found between ELISA and CIE tests; 99.7% with *A. suum* adult worm’s antigen (676 out of the 678 negatives for both techniques) and 99.9% with *TES* (677 out of the 678 negatives for both techniques).

Lower results were obtained in predictive positive values, with 44.7% and 18.2% for *A. suum* and TES, respectively.

### 3.2. Distribution along the Decade

Over the decade, an average of 77 samples/year were received as suspected of larva migrans, recording a maximum of 123 samples/year in 2010 and a minimum of 47 samples/year in 2020.

**Figure 1:**
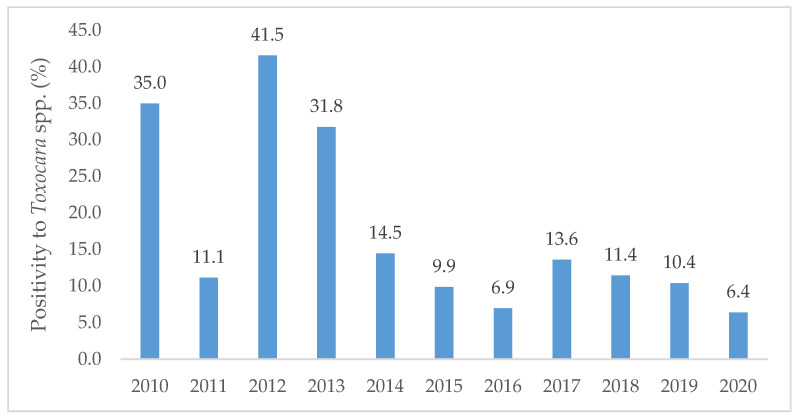
Percentage of positive samples to *Toxocara* spp. per year along the decade from 2010 to 2020, diagnosed at the Institute of Hygiene and Tropical Medicine.

The year of 2012 registered the highest positivity (41.5%, *n* = 27/65) followed by 2010 (35.0%, *n* = 43/123) and 2013 (31.8%, *n* = 27/85). A minimum positivity was noted in 2020 (6.4%, *n* = 3/47). A general decreasing trend in the number of positives was found from 2010 to 2020 (Figure 1).

### 3.3. Distribution by Age and Sex

**Figure 2:**
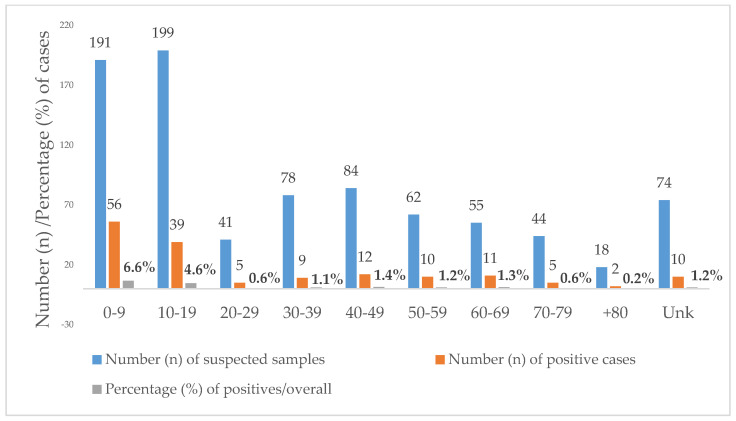
Age distribution of *Toxocara* positive cases diagnosed at the Institute of Hygiene and Tropical Medicine (IHMT) from 2010 to 2020. Unk = unknown age.

Toxocariasis was equally prevalent in females (18.8%, *n* = 66/351) and males (18.8%, *n* = 93/495). Regarding the age of the positives, it ranged from 1 to 85 years. Overall, 46.1% (390/846) of the suspected samples were requested from individuals under the age of 20. Additionally, 59.7% (95/159) of the positives were diagnosed in children younger than 20 years, i.e., 35.2% (56/159) in children aged 0–9 years and 24.5% (39/159) in teenagers aged 10–19 years. Positivity was higher in the age groups below 20 years with a statistically significant difference between the age group 0–9 years (29.3%) and the age group 10–19 years (19.6%; *p* < 0.012) (Figure 2).

The median age of diagnosis of toxocariasis was 24 years, more precisely, 26 for females and 22 years for males. If we divide the samples into two main age groups, children (aged 0–19 years) and adults (older than 19 years), we will find a positivity for *Toxocara* infection of 24.4% (95/390) in children and positivity of 14.1% (54/382) in adults, with significant statistical differences between age groups (χ^2^, *p* < 0.001).

Age was unknown in 74 cases, of which 10 were positive.

### 3.4. Distribution by Provenance of the Samples

**Figure 3:**
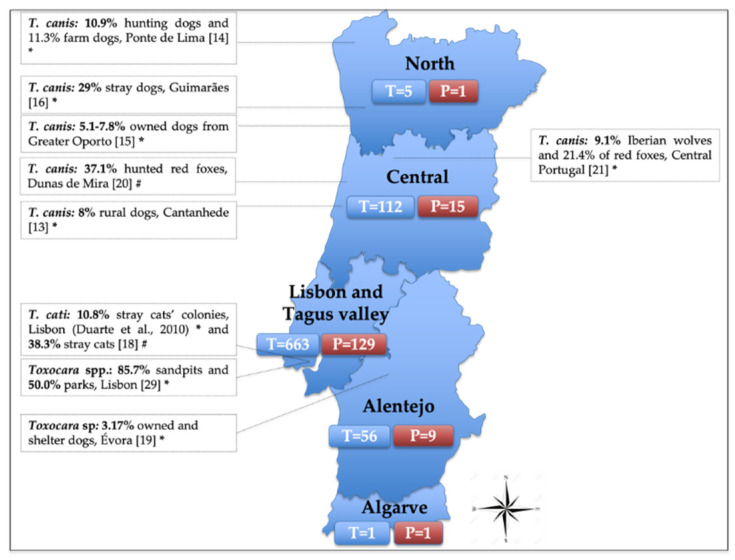
Distribution of the tested (T) and positive (P) human samples for *Toxocara* spp., received at the Institute of Hygiene and Tropical Medicine (IHMT) from 2010 to 2020, divided by its provenance according to the five regional health units of continental Portugal. T = number of tested samples (nine samples are not shown in the figure as provenance is unknown); P = number of positive samples (four positives are not shown in the figure as provenance is unknown). The side text boxes show the epidemiological studies conducted to assess *Toxocara* prevalence in soil and in animal species in Portugal. * Samples tested by coprological analysis; # Samples tested by parasitological necropsy.

Regarding the provenance, the five regional health units of continental Portugal recorded positive cases for *Toxocara* spp. Out of the health units with five or more tested samples, North registered the highest positivity (20%, 1/5), followed by LTV (19.5%, 129/663), Alentejo (16.1%, 9/56) and Central (13.4%, 15/112) (Figure 3). Most of the samples came from LTV as the IHMT location is in Lisbon. Unknown provenance was reported in nine samples, of which four were positive and five were negative.

### 3.5. Distribution by Medical Specialties

Regarding the medical specialties, Pediatrics (*n* = 275), Infectiology (*n* = 210) and Internal medicine (*n* = 62) were those who most required an analysis of a potential clinical presentation of toxocariasis. Pediatrics was also the specialty with the highest percentage of positives (41.5%, 66/159), followed by Infectiology (25.8%, 41/159) and Internal medicine (5.7%, 9/159) (Table 1).

### 3.6. Features Reported by Clinicians

Despite the detected percentage of exposure to *Toxocara* spp., a low number of people developed larva migrans and/or clinical manifestations. Peripheral blood eosinophilia was the most frequent feature reported by clinicians for the analysis of the suspected samples. Overall, it was detected in 44 out of the 159 positive samples (27.7%) and in 5.2% of overall samples. Additional findings detected in positive samples are presented in Table 2.

Out of the 152 positives for *Toxocara* spp., only two referred had close contact with cats/dogs and only one was referred with consumption of raw/undercooked meat.

## 4. Discussion

Overall, out of the 846 samples requests for human *Toxocara* serology, a positivity of 18.8% was found, with positives detected throughout continental Portugal. This value is close, although slightly inferior, to the 21.9% reported from an official laboratory (INSA) in southern Portugal from 1995 to 2005 in 457 suspected human samples [12]. No seroprevalence surveys have been performed so far in humans in Portugal, which together with the absence of systematically collected data, makes comparison quite difficult. Nevertheless, reports from neighboring European countries looking for *T. canis* IgG antibodies with ELISA show seroprevalences of 8.0% in residents of Catania, Italy [24] and 16.0% in the residents from Attica, Greece [25]. A recently published systematic review and meta-analysis revealed a 19.0% global seroprevalence of toxocariasis in human populations, ranging from 8.2% in the Eastern Mediterranean to 37.7% in Africa, with a pooled seroprevalence of 10.5% (8.5–12.8%) in the European region [26]. As found by Rostami et al., 2019 [26], higher seroprevalences of *Toxocara* were associated mainly with economic and environmental factors, such as lower-income level, lower human development index, lower latitude, higher humidity, higher temperature and higher precipitation.

Based on the tested and positive samples received at IHMT, toxocariasis seems to be decreasing in Portugal over the last decade. Some factors may account for that, such as the growing use of broad-spectrum anthelmintic in pets, educational campaigns/fines to promote feces collection by the owners, stray dogs’ control, the replacement of children’s sandpit playgrounds for synthetic floors and the vast construction of dog parks by several municipalities. Other aspects that may explain such a low number of suspected samples and diagnosed cases of toxocariasis in 2020 might be related to the avoidance or delay of medical care regarding the pandemic of SARS-COV-2.

It is estimated that ≥100 million dogs are infected with *Toxocara* around the world, with a global prevalence in dogs of 11.1% (95% CI, 10.6–11.7%), and an estimated prevalence in Europe of 10.8% (8.9–12.9%) [27]. Precedent data regarding the prevalence of *Toxocara* spp. in animals show a high prevalence and wide distribution throughout Europe [28]. In Portugal, the situation is similar, not only in domestic carnivores like cats and dogs but also in wildlife species, such as the Iberian wolf and the red fox (Figure 3). However, as most surveys on carnivores were exclusively based on a fecal examination, they may not accurately reflect the true prevalence of *Toxocara* spp. in the country, given some of their inherent limitations (i.e., lack of sensitivity with low parasite burdens, intermittent shedding, or absence of parasite shedding during the pre-patent period or immature infections). Nevertheless, such prevalence of *Toxocara*-infected carnivores serves as rich sources of eggs to the environment.

Urban areas, particularly public parks of Lisbon have shown to be heavily contaminated with *Toxocara* spp. eggs [29]. From the 151 soil samples and 135 canine fecal samples collected in the city, 85.7% of the sandpits and 50.0% of the parks were contaminated with *Toxocara* spp. eggs. Additionally, an average density of 4.2 eggs per hundred grams of soil was found and 56.0% of the recovered eggs were embryonated after 60 days of incubation, therefore, were considered viable and infective. These results are concerning as the studied areas represent common places where people of all ages, including children, recreate. Furthermore, close physical contact between Portuguese owners and their dogs is frequent: in 43.1% of the households, dogs were allowed to sleep with the owners in their beds and in 75.5% to lick their owners’ faces [30]. Although close contact between pets and humans is mutually beneficial, these reported behavioral practices can also increase the risk of transmission of zoonotic diseases. In fact, to the best of the author’s knowledge, very few cases of *Toxocara* spp. infection in humans have been reported in Portugal. There is a case of a panuveitis caused by an ocular infection with *T. canis* in a 9-year-old boy presented with sudden unilateral vision loss at the service of Ophthalmology, in Lisbon [31], and a case of acute pericarditis in a child caused by *T. canis* infection presented at the service of Pediatric cardiology, in Porto [32]. Actually, the true number of cases occurring in Portugal are probably underestimated. Several factors may account for that, including the fact that most infections are asymptomatic and clinical investigation and diagnostic testing are frequently not conducted [7,33]. Moreover, the laboratory diagnosis of toxocariasis is still relatively insensitive, especially for ocular cases where serological tests may present as false negatives [10]. Furthermore, as toxocariasis is a relatively rare infection, its diagnosis requires a professional with knowledge of its symptoms and epidemiology. Nevertheless, it should be highlighted a vast number of medical specialties (*n* = 20) are suspected of *Toxocara* exposure in the current study.

In our study, exposure to *Toxocara* spp. was equally common in females and males, in opposition with the bibliography that reports male sex as a potential risk factor associated with seropositivity to *Toxocara* spp. [26]. Additionally, 59.7% of the positives were diagnosed in children younger than 20 years, in line with the bibliography that reports toxocariasis as a predominant disease of children, given their pica behaviors and poor hand hygiene [26]. In a study conducted to assess parasite control practices in owned pets from Lisbon (Portugal), although 89.7% of the dogs and 63.6% of the cats were being treated with endoparasitic drugs, merely 11.8% of the dogs and 5.5% of the cats were treated with the recommended regimen (minimum quarterly) [34,35]. This report showed that although the majority of pet owners give antiparasitic drugs, most of them do not follow the manufacturer’s recommendations, deworming at irregular and consequently ineffective intervals [35]. Therefore, periodic deworming of household pets, in general, should be promoted, especially puppies and kittens along with pregnant bitches/cats which are more prone to transmit the disease. Besides, as found by Matos et al., 2015 [35], 37% of Portuguese dog owners did not collect their dog’s feces in all public places, claiming several excuses: feces considered fertilizers (43.2%); feces located on abandoned/unreachable areas (37.7%); laziness/repulsiveness (14.5%) and shame (4.6%). Owners should safely collect and hygienically dispose of their pet’s feces, avoiding eggs becoming infective and consequently breaking the dog-soil-human transmission cycle of toxocariasis. Additionally, the lack of an effective anthelmintic method to kill *Toxocara* spp. eggs make it difficult to eradicate this parasite from the environment [36]. Taking into account the potential impact of not collecting feces and the potential zoonotic nature of some of the pathogens, this is regrettable and highlights the need for owner awareness to reduce environmental contamination pressure and safeguard public and animal health. Preventing environmental contamination through rigorous fecal removal practices should be encouraged, along with effective strategies to prevent human infections, such as precluding pets and stray animals from accessing children’s play areas, feral and stray pet populations control, fencing playgrounds, covering, sterilizing, or even eliminating sandpits from public zones. Likewise, discouraging geophagia in children is crucial along with the promotion of handwashing after playing, or touching pets, or following exposure to potentially contaminated sites. Additionally, according to Matos et al., 2015 [35], 85% of the pet owners inquired had never heard of the word “zoonosis” and one-third were unable to cite any possible parasitic infection sources.

Concerning the clinical symptoms referred by the clinicians, most of the positives had no associated findings, which is coherent with a bibliography that shows that human toxocariasis is mostly asymptomatic [37]. Peripheral blood eosinophilia was indeed the most frequent feature reported, detected in more than one-quarter of the positive samples.

Some limitations of the study should be emphasized. Although ELISA is widely used, is not the gold standard test. ELISA could generate false-positive results due to cross-reactivity with other helminths (especially *A. lumbricoides)* and it has a lack of ability to differentiate between active and past infections. Therefore, a positive serology does not mark an infection but a potential serological scar and no tool is currently available to distinguish between the two [38]. For that reason, whenever available and economically feasible, serological screenings should be complemented with other methods, such as detection of anti-*Toxocara* antibodies by Western blot using TES antigens, to increase the accuracy of the diagnosis and to establish the implicated species. Another limitation was the lack of clinical information, such as clinical symptoms and *Toxocara*-associated risk factors. This absence of information not only jeopardizes crucial epidemiological data as easily explains why such a low number of positives had a history of cat/dog/soil contact or undercooked meat consumption. On the other hand, it might show that many physicians are not fully knowledgeable about *Toxocara* spp. cycle and routes of infection. Finally, the lack of a multi-sectoral and multi-institutional cooperation net between distinct laboratories precludes an effective surveillance infrastructure, hardening the estimation of disease burden in the country.

## 5. Conclusions

This is the most comprehensive study carried out in Portugal to assess the occurrence of this neglected zoonosis in the country. An overall positivity of 18.8% of human toxocariasis was found, indicating that toxocariasis continues to be a health problem, particularly in children. Effective preventive measures and public awareness should be encouraged to minimize the health-risk impact on both animals and humans. An integrated multidisciplinary ‘One Health’ approach strengthens partnerships among local authorities, physicians, veterinarians and policymakers is crucial to achieving a joint action and better control of this zoonotic disease.

## Figures and Tables

**Table 1:** Number of tested samples and positive samples for *Toxocara* spp. according to the requested medical specialty from 2010 to 2020.

Specialty	Tested Samples (*n*)	Positive Samples (*n*)	Positivity (%)
Pediatrics	275	66	41.5
Infectiology	210	41	25.8
Internal medicine	62	9	5.7
Travel medicine	53	6	3.8
Hematology	15	6	3.8
Pulmonology	56	5	3.1
Gastroenterology	23	5	3.1
Orthopedics	32	2	1.3
Ophthalmology	18	2	1.3
Pediatric cardiology	2	2	1.3
Clinical pathology	21	1	0.6
Rheumatology	8	1	0.6
Immunoallergology	7	1	0.6
Pediatric surgery	2	1	0.6
Neurology	7	0	0
Dermatology	1	0	0
Nephrology	1	0	0
Otorhinolaryngology	1	0	0
Psychiatry	1	0	0
Radiation oncology	1	0	0
Unknown	50	11	6.58
Total	846	159	18.8

**Table 2:** Clinical manifestations were referred by the clinicians according to the tested samples and positive samples for *Toxocara* spp. Positivity shows the percentage of cases with clinical manifestation over the 846 samples tested.

Clinical Manifestations	Tested Samples (*n*)	Positive Samples (*n*)	Positivity/Overall (%)
Peripheral blood eosinophilia	167	44	5.2
Hepatic nodule(s)	10	6	0.7
Hepatosplenomegaly/splenomegaly/hepatomegaly	13	5	0.6
Fever	19	5	0.6
Elevated level of total immunoglobulin E	14	4	0.5
Unilateral uveitis/retinitis/chorioretinitis	18	4	0.5
Cough/wheezing	8	3	0.4
Abdominal pain	10	3	0.4
Pruritus/skin rash/dermatitis	19	3	0.4
Convulsions	1	1	0.1
Bronchospasm	4	1	0.1
Weight loss	10	1	0.1
Persistent diarrhea	21	1	0.1
Suspected cerebral lesion(s)	7	0	0
Emesis	5	0	0
Lymphadenopathy	5	0	0
Pulmonary eosinophilia	5	0	0
Central nervous system infection	2	0	0
**Habits**			
Cat/dog contact	11	2	0.2
Undercooked meat consumption	4	1	0.1
Soil contact	2	0	0
